# Causal Relationship Between the Risk Factors and Work-Related Musculoskeletal Disorders Among Professional Drivers: A Systematic Review

**DOI:** 10.1177/00187208211006500

**Published:** 2021-06-14

**Authors:** Leonard Joseph, Lenny Vasanthan, Miles Standen, Raija Kuisma, Aatit Paungmali, Ubon Pirunsan, Patraporn Sitilertpisan

**Affiliations:** 1 156588 University of Brighton, East Sussex, UK; 2 30025 Christian Medical College, Vellore, Tamil Nadu, India; 3 52912 North Karelia University of Applied Sciences, Joensus, Finland; 4 26682 Chiang Mai University, Thailand

**Keywords:** musculoskeletal disorders, ergonomics, risk assessment, risk factors, professional drivers

## Abstract

**Objective:**

This review evaluates the evidence on the strength of causal relationship between categories of risk factors (RFs) and work-related musculoskeletal disorders (WRMSDs) among professional drivers.

**Background:**

A compilation of evidence on the causal relationship between RFs and WRMSDs among professional drivers is lacking.

**Methods:**

A systematic search of the literature was conducted in major electronic data bases that include Medline (1946 + via OvidSP), Embase (1974 + OvidSP), CINAHL (1982+), AMED, and Web of Science. The methodological quality of the studies was assessed and scored. A descriptive analysis on the categories of RFs associated with WRMSDs was conducted. The Bradford–Hill causation criteria and evidence interpretation tool were used to evaluate the causal relationship between RFs and WRMSDs in professional drivers.

**Results:**

Among the 54 studies reviewed, a strong evidence suggests a causal relationship between RFs such as whole-body vibration, awkward postures, lifting tasks, manual material handling, job stress, job demand, and previous pain episodes with WRMSDs. Moderate evidence was observed on RFs such as uncomfortable seat and low job satisfaction. The evidence on causal relationship between RFs such as years of professional driving, driving duration, and individual characteristics such as age and body mass index was inconclusive.

**Conclusion:**

There is strong to moderate evidence on the causal relationship between the physical and psychosocial RFs and WRMSDs among professional drivers.

**Application:**

Potential application of this review highlights evidence to occupational health practitioners, policy makers, and stakeholders on the strength of causal relationship between RFs and WRMSDs among professional drivers.

## Introduction

Work-related musculoskeletal disorders (WRMSDs) are syndromes of the musculoskeletal system such as bones, muscles, joints, and tendons which occur due to work- or work-related environment (Sekkay et al., 2018). Professional driving is an occupation in the transport sector with high rates of WRMSDs (Health & Safety Executive [HSE], 2015). Professional driving is defined as an occupation that requires a person to drive a motorized vehicle as an occupational task for a long period of time (Tamrin et al., 2014). Professional drivers often face severe adverse conditions such as traffic congestion, continuous time pressure, excessive physical demands, and so on, which could challenge their health condition and expose them to WRMSDs (Montoro et al., 2018). A recently published report by the US Bureau of Labor Statistics shows that bus drivers as professional drivers are one of the top three occupations that has the highest rates of musculoskeletal disorders with highest incidence rates (206 per 10,000 full-time workers; the US Bureau of Labor Statistics, 2017). Also, recent evidence shows that the prevalence of WRMSDs in bus drivers, truck drivers, and taxi drivers is 80%, 81%, and 71%, respectively (Geete et al., 2013; Mozafari et al., 2015; Robb & Mansfield, 2007).

Epidemiological evidence suggests that WRMSDs are caused by three types of risk factors (RFs): physical, psychosocial, and individual (National Research Council (US) and Institute of Medicine (US) Panel on Musculoskeletal Disorders and the Workplace, 2001). The physical RFs contributing to WRMSDs include prolonged sitting, exposure to whole-body vibration (WBV), static or awkward postures, continuous movements, excessive forces, lack of recovery between the movements, and repetitive actions (Bovenzi, 2009; Krause et al., 1998; Sekkay et al., 2018). The psychosocial RFs associated with WRMSDs among professional drivers are stress, low job satisfaction, and job demand, although the magnitude of evidence varies across different studies and designs (Hoogendoorn et al., 2000; Krause, Ragland, Greiner, Syme, et al., 1997; Lötters et al., 2003). Certain individual RFs such as age, body mass index (BMI), general health status, and previous symptoms have also been linked to WRMSDs (da Costa & Vieira, 2010; Lyons, 2002). To lessen the incidence of WRMSDs associated with professional driving, it is necessary to improve our understanding on the RFs that contribute to the development of WRMSDs.

The majority of the available scientific evidence has merely reported on the prevalence of WRMSDs among professional drivers (Alperovitch-Najenson, Katz-Leurer, et al., 2010; Chen et al., 2004; Szeto & Lam, 2007). While individual studies had reported on the RFs for WRMSDs (Andrusaitis et al., 2006; Anjomshoae & Abdul Rani, 2013; Burgel & Elshatarat, 2017), scientific evidence on the comprehensive evaluation of the strength of the causal relationship between RFs and WRMSDs among professional drivers was lacking. While the policies in the transport sector mainly focus on infrastructure investments, pricing incentives, and regulatory aspects such as carbon emission, both guidelines to promote health and well-being and effective policies to prevent WRMSDs among professional bus drivers remain deficient in this sector (Berg et al., 2017). Thus, a significant knowledge gap and a need for a comprehensive review were identified and served as a motivation to the Sustained Model of Assessment and Rehabilitation Training (SMART Drive) network group, which comprises an international group of researchers in the field of occupational health and WRMSDs.

The aim of this systematic review was to evaluate the evidence on the strength of causal relationship between categories of RFs and WRMSDs among professional drivers. Therefore, this review focused on a key research question: What is the current evidence on the strength of the causal relationship between RFs and WRMSDs among professional drivers? A comprehensive and thorough understanding of the causal relationship between RFs and WRMSDs among professional drivers might be beneficial to establish professional guidelines and policies for the management of WRMSDs.

### Methods

This systematic review was conducted and reported according to the PRISMA (Preferred Reporting Items of Systematic Reviews and Meta-Analyses) guidelines for reporting systematic review findings (Liberati et al., 2009; Moher et al., 2015).

### Literature Search

A comprehensive electronic search of Medline (1946 + via OvidSP), Embase (1974 + OvidSP), CINAHL (1982+), AMED, PubMed, and Web of Science was conducted from the year 1980 until the year May 2018. MeSH and key text word search terminologies on professional driving, musculoskeletal disorder, and RFs were used to search the literature using the Boolean operators in all of the above-named databases. A full search strategy used in the Embase database is presented in Appendix A. Reference lists of included studies and a gray literature search was conducted using the following sources of information: Open Gray, The King’s Fund, and WHO (World Health Organization).

### Eligibility Criteria

Studies were included in the review using the following criteria: (1) professional drivers >18 years with at least 1 year of professional driving experience; (2) professional drivers, defined as those who drive as a full-time occupation; (3) all types of professional drivers reported in the literature, which includes bus, truck, car/taxi, minibus, van, forklift, tractor, crane, and heavy equipment machinery; (4) studies that examined the RFs associated with WRMSDs among professional drivers; (5) studies published in peer-reviewed English-language journals; (6) methods utilized include cross-sectional, case-control, or prospective cohort study designs; (7) results were reported separately on RFs for WRMSDs associated with professional driving. The studies were excluded from the review if they (1) had no specific population (e.g., too broad); (2) were nonscientific studies (e.g., editorials, commentaries) or literature reviews; and (3) were related only to treatment of pain, basic sciences, or cadaver studies.

### Screening Process

Once the studies were identified, they were exported into Endnote to check for duplication. Duplicated studies were removed accordingly. Bibliographic records were then exported from Endnote into Microsoft Excel to enable further manual deletion of duplications. Initial screening was conducted first on the title and abstract by one review author and cross-examined by the second review author. Second-level screening evaluated full-text reports of studies deemed potentially eligible. Any disagreements between reviewers were resolved by discussion and reflection.

### Methodological Quality Assessment

Two reviewers (MS and LJ) used three types of quality assessment tools to examine the methodological quality of the cross-sectional, case-control, and prospective cohort studies, respectively. The quality of the cross-sectional studies was assessed using the Hoy et al. (2012) risk of bias tool. The tool assesses the external validity through four items (1–4) and evaluates the internal validity using six items (5–10), which provides an overall methodological quality score. The case-control version of the Newcastle–Ottawa Scale was used to evaluate the case-control studies and the Newcastle–Ottawa quality assessment scale for the cohort studies was used for prospective studies, both of which assess nine items of selection, comparability, and outcome (Stang, 2010). Total scores from the risk of bias tool and Newcastle–Ottawa scales were categorized into three groups: very high risk of bias (0–4 points), high risk of bias (5–6 points), and low risk of bias (7+ points; Lo et al., 2014). The overall methodological quality of the studies was rated using the following classifications: high quality (low risk of bias), medium quality (high risk of bias), and low quality (very high risk of bias). This method is consistent with the Grades of Recommendation, Assessment, Development and Evaluation (GRADE) and Cochrane approaches (Guyatt et al., 2008).

### Data Abstraction

The study characteristics extracted from the reviewed studies included authors, year of publication, country of study, aim of study, study design, the types of RFs studied, type of vehicle, and number and mean age of the participants. Where available, the information related to the RFs such as the values of Pearson and Spearman’s correlations (*r*); measures of association such as reported odds, risk, or hazard ratios (ORs, RRs, or HRs, respectively); and confidence intervals (CIs) and/or *p* value were extracted and used for review.

### Analysis and Level of Evidence

A descriptive analysis was conducted to report the RFs. The statistical assessment for specific Bradford–Hill criteria for causation was used to evaluate the strength of relationship between WRMSDs and associated RFs and the effect sizes were interpreted (Roffey et al., 2010; Rosenthal, 1996). A brief description of the Bradford–Hill criteria for causation and the interpretation of the strength of relationship is shown in Table 1. This review focused on the size and direction of the risk estimate, irrespective of the level of significance. A reported nonsignificant association between a risk factor and WRMSD, with no mention of risk estimate or direction of association, was disregarded as it would not be clear whether the risk estimate was increased or decreased. Reporting a significant association without presenting a risk estimate was considered as a finding and contributed to the level of evidence. For those studies reporting an association, unadjusted results were presented if multivariate results had not been calculated. Risk estimates were presented with CIs (if reported) for each study reporting an association. The description and strength of the levels of evidence relating to the Bradford–Hill criteria is presented in Table 2 (Roffey et al., 2010; Rosenthal, 1996).

**Table 1:** Statistical Assessment for Specific Bradford–Hill Criteria for Causation

Criteria Assessed	Statistical Measures	Interpretation of Strength of Relationship
Association and experiment	Odds ratio	Protective: <1.0 Weak: 1.0–2.4 Moderate: 2.5–3.9 Strong: >4.0
	Relative risk, hazard ratio, prevalence ratio, incidence rate ratio	Protective: <1.0 Weak: 1.0–1.9 Moderate: 2.0–2.9 Strong >3.0
	*T* test	Clinically significant: >10% change in effect
Consistency of findings	Sackett’s strength of evidence	Strong: >75% of studies (at least 2 high quality)
Dose response relationship	Pearson correlation	Protective: <.0 Weak: 0.1–0.29 Moderate: 0.3–0.49 Strong: >.5
	Logistical regression	Protective: <.0 Weak: 0.1–0.29 Moderate: 0.3–0.49 Strong: >.5
	Confidence intervals on estimates	Significant: nonoverlapping Trend: overlapping confidence interval
If a nonsignificant association was reported between a risk factor and WRMSD, it was categorized as “no association.”		

*Note.* ^*^Strength refers to how strong a relationship is for the unique risk estimate (Roffey et al., 2010). WRMSD = work-related musculoskeletal disorders.

**Table 2:** Description of Levels of Evidence Relating to the Bradford–Hill Criteria

Description of Evidence	Evidence Strength
2 or more high-quality prospective cohort studies with consistent multivariate results.	Strong evidence
1 high-quality study or 2 low-quality prospective cohort studies with consistent multivariate results	Moderate evidence
1 low-quality prospective cohort study of unadjusted results.	Limited evidence
Inconsistent studies of the same quality (consistent high quality >inconsistent low quality), or consistent findings in low-quality cross-sectional studies.	Inconclusive evidence

*Note.* Roffey et al. (2010).

## Results

### Study Characteristics

**Figure 1:**
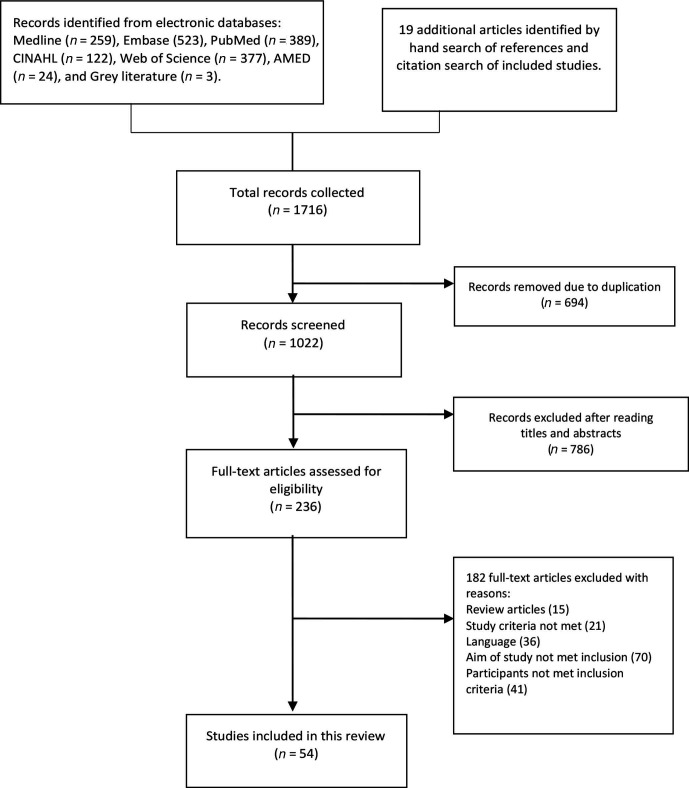
Flowchart of the study selection process.

A total of 54 studies were included in the review. Figure 1 shows the flowchart of the selection procedure.

Among the 54 included studies that showed evidence on the relationship between WRMSDs and RFs among the professional drivers, three main categories of RFs were identified: physical, psychosocial, and individual. The RFs that did not fall under these three categories were grouped separately as “other RFs.” The various physical RFs reported among the studies were WBV exposure (20 studies), awkward posture (16 studies), static postures (4 studies), lifting tasks (11 studies), bending and twisting (4 studies), and manual material handling (6 studies). Perceived job stress (11 studies), perceived job demand (9 studies), low job satisfaction (12 studies) and effort–reward balance (2 studies) were the different psychosocial RFs reported by studies among professional drivers. Age (31 studies), weight/BMI (27 studies), lack of physical/sporting activity (17 studies), history of previous musculoskeletal pain episodes (29 studies), smoking (13 studies), drinking alcohol (7 studies), lower levels of education (12 studies), and female gender (7 studies) were reported as individual RFs. RFs such as years in driving occupation (23 studies), driving duration (day/week; 28 studies), and uncomfortable seat (9 studies) and decision latitude (7 studies) were grouped as other RFs. The different types of vehicle reported in various studies include bus (27 studies), truck (16 studies), car/taxi (12 studies), forklift (4 studies), tractor (2 studies), minibus (2 studies), van (2 studies), crane (2 studies), and straddle carrier (1 study).

### Overall Quality of the Reviewed Studies

Among the 54 studies that investigated the relationship between RFs and WRMSDs in professional drivers, 39 were cross-sectional studies, four were case-controlled studies, and 11 were prospective studies. The overall methodological quality varied from low (5 studies), moderate (30 studies), and high (19 studies). Among the 39 cross-sectional studies, 26 were of moderate quality, 11 of high quality, and three of low quality. In terms of quality evaluation of the studies, “the selection of the study population in individual studies” (criteria 1) was not well defined in most of the cross-sectional studies (Table 3).

Table 3 shows the individual scores of all the cross-sectional studies included in this review.

**Table 3:** Methodological Quality Scores of Cross-Sectional Studies

Author (Year)	Types of Vehicle Studied	External Validity				Internal Validity						Quality
		1	2	3	4	5	6	7	8	9	10	
Abledu et al. (2014a)	Minibus	N	N	N	N	Y	N	Y	Y	Y	Y	(5)Med
Abledu et al. (2014b)	Taxi	N	N	N	N	Y	N	Y	Y	Y	Y	(5)Med
Akinpelu et al. (2011)	Cars & minibus	N	N	N	N	Y	N	Y	Y	Y	Y	(5)Med
Alperovitch-Najenson, Katz-Leurer, et al. (2010)	Bus	N	N	Y	N	Y	N	Y	Y	Y	Y	(6)Med
Alperovitch-Najenson, Santo, et al. (2010)	Bus	N	N	Y	N	Y	N	Y	Y	Y	Y	(6)Med
Aminian et al. (2016)	Truck and taxi	N	N	N	Y	Y	N	Y	Y	Y	Y	(6)Med
Andrusaitis et al. (2006)	Truck	N	N	N	N	Y	Y	N	Y	Y	Y	(5)Med
Anjomshoae and Abdul Rani (2013)	Bus	N	N	N	N	Y	N	Y	Y	Y	Y	(5)Med
Boshuizen et al. (1990)	Tractor	N	N	N	N	Y	Y	N	Y	Y	Y	(5)Med
Boshuizen et al. (1992)	Truck	N	N	N	N	Y	Y	N	Y	Y	Y	(5)Med
Bovenzi et al. (2006)	Truck, forklifts, cranes, and bus	Y	Y	Y	N	Y	Y	Y	Y	Y	Y	(9)High
Bovenzi and Betta (1994)	Tractor	N	N	N	N	Y	N	N	N	Y	Y	(3)Low
Bovenzi and Zadini (1992)	Bus	N	N	N	Y	Y	N	Y	N	Y	Y	(5)Med
Burdorf et al. (1993)	Straddle carrier	N	N	N	N	Y	Y	Y	Y	Y	Y	(6)Med
Burgel and Elshatarat (2017)	Taxi	N	N	N	N	Y	N	Y	Y	Y	Y	(5)Med
Chen et al. (2004)	Taxi	N	N	N	Y	Y	N	Y	Y	Y	Y	(6)Med
Chen et al. (2005)	Taxi	N	N	N	Y	Y	N	Y	Y	Y	Y	(6)Med
Feng et al. (2020)	Taxi	Y	Y	N	N	Y	Y	Y	Y	Y	Y	(8)High
Gangopadhyay and Dev (2012)	Bus	N	N	N	N	Y	N	Y	Y	Y	Y	(5)Med
Geete et al. (2013)	Bus	N	N	N	N	Y	N	N	Y	N	Y	(3)Low
Greiner and Krause (2006)	Bus	N	Y	Y	Y	Y	Y	N	Y	Y	Y	(8)High
Gyi and Porter (1998)	Police car	N	N	Y	N	Y	N	Y	Y	Y	Y	(6)Med
Krause, Ragland, Greiner, Fisher, et al. (1997)	Bus	N	N	N	Y	Y	Y	N	Y	N	Y	(5)Med
Miyamoto et al. (2008)	Taxi	N	N	N	N	Y	N	Y	Y	Y	Y	(5)Med
Miyamoto et al. (2000)	Truck	N	N	N	N	Y	N	N	Y	Y	Y	(4)Low
Nazerian et al. (2020)	Crane	Y	Y	N	Y	Y	Y	Y	Y	Y	N	(8)High
Okunribido et al. (2006)	Van	N	N	Y	N	Y	N	Y	N	Y	Y	(5)Med
Okunribido et al. (2008)	Bus, car, & van	N	N	Y	N	Y	N	Y	N	Y	Y	(5)Med
Okunribido et al. (2007)	Bus	N	N	Y	N	Y	N	Y	N	Y	Y	(5)Med
Porter and Gyi (2002)	Car	Y	Y	Y	N	Y	N	Y	Y	Y	Y	(8)High
Raanaas and Anderson (2008)	Taxi	Y	Y	N	N	Y	N	Y	Y	Y	Y	(7)High
Robb and Mansfield (2007)	Truck	N	Y	Y	N	Y	N	Y	Y	Y	Y	(7)High
Rugbeer et al. (2016)	Bus	N	N	N	N	Y	N	Y	Y	Y	Y	(5)Med
Sekkay et al. (2018)	Truck	Y	N	N	Y	Y	Y	Y	Y	Y	Y	(8)High
Senthanar and Bigelow (2018)	Truck	Y	Y	N	N	Y	Y	Y	N	Y	Y	(7)High
Szeto and Lam (2007)	Bus	N	Y	N	N	Y	N	Y	Y	Y	Y	(6)Med
Tamrin et al. (2007)	Bus	N	Y	N	N	Y	N	Y	Y	Y	Y	(6)Med
Tamrin et al. (2014)	Bus	N	Y	Y	N	Y	N	Y	Y	Y	Y	(7)High
Wang et al. (2017)	Bus	N	Y	Y	N	Y	N	Y	Y	Y	Y	(7)High

*Note.* N = no; Y = yes; High = high quality (low risk of bias); Med = medium quality (moderate risk of bias); Low = low quality (high risk of bias); 1 – Was the study’s target population a close representation of the national population in relation to relevant variables, age, sex, occupation? 2 – Was the sampling frame a true or close representation of the target population? 3 – Was some form of random selection used to select the sample, OR, was a census undertaken? 4 – Was the likelihood of non-response bias minimal? 5 – Were data collected directly from the subjects (as opposed to a proxy)? 6 – Was an acceptable case definition used in the study? 7 – Was the study instrument that measured the parameter of interest (e.g. prevalence of low back pain) shown to have reliability and validity (if necessary)? 8 – Was the same mode of data collection used for all subjects? 9 – Was the length of the shortest prevalence period for the parameter of interest appropriate? 10 – Were the numerator(s) and denominator(s) for the parameter of interest appropriate?

There were three moderate quality studies and one low quality study among the case-control studies. The overall quality of the prospective studies was high (8 studies) in addition to two moderate quality studies and one low quality study. Tables 4 and 5 show the quality of the case-controlled and prospective studies that reported the relationship between RFs and WRMSDs among professional drivers.

**Table 4:** Methodological Quality Scores of Case-Control Studies Tool

Author (Year)	Types of Vehicle Studied	Overall Items									Quality
		1	2	3	4	5	6	7	8	9	
Anderson (1992)	Bus	Y	Y	Y	Y	N	N	N	Y	Y	(6)Med
Magnusson et al. (1996)	Truck and bus	N	N	N	N	N	N	N	Y	Y	(2)Low
Mozafari et al. (2015)	Truck	Y	N	Y	Y	Y	N	N	Y	N	(5)Med
Palmer et al. (2008)	Car, bus, truck, & forklift	Y	N	Y	Y	Y	N	N	Y	N	(5)Med

*Note.* N = no; Y = yes; Med = medium quality (moderate risk of bias); Low = low quality (high risk of bias); 1 – Case definition. 2 – Representation of cases. 3 – Selection of controls. 4 – Definition of controls. 5 – Study controls for important factor. 6 – Study controls for an additional factor. 7 – Ascertainment of exposure. 8 – Same method of ascertainment used for both cases and controls. 9 – Non-response rate.

### Physical Risk Factors

The findings supported strong evidence demonstrating a weak relationship between WRMSDs and WBV among professional drivers. Of 20 studies reviewed, 19 were consistent with demonstrating a positive relationship with ORs ranging from 1.3 to 4.9. The results showed strong evidence that awkward postures (such as nonneutral postures, bending-turning the torso, twisting the neck) had a weak relationship with WRMSDs among professional drivers. Of 15 studies reviewed, 12 were consistent in demonstrating a positive relationship with ORs ranging from 1.6 to 3.2. Also, there was strong evidence for a weak relationship between WRMSDs and lifting tasks in professional drivers. Of 11 studies reviewed, nine were consistent in demonstrating a positive relationship with ORs ranging from 1.2 to 2.4. Only one study (Sekkay et al., 2018) reported a relationship between a risk factor “doing work that requires forceful exertion” and WRMSDs with ORs 2.96 (1.39–6.26). Also, there was strong evidence for a moderate relationship between WRMSDs and manual material handling. Table 6 shows the list of the physical RFs and its relationship with WRMSDs among professional drivers.

**Table 5:** Methodological Quality Scores of Prospective Cohort Studies Tool

Author (Year)	Types of Vehicle Studied	Overall Items									Quality
		1	2	3	4	5	6	7	8	9	
Bovenzi (2009)	Truck and bus	Y	Y	Y	N	Y	Y	N	Y	Y	(7)High
Bovenzi (2010)	Truck and bus	Y	Y	Y	Y	Y	Y	N	Y	N	(7)High
Bovenzi (2015)	Truck, forklift, and bus	Y	Y	Y	N	Y	Y	N	Y	Y	(7)High
Bovenzi, Schust, et al. (2015)	Truck and bus	Y	Y	Y	N	Y	Y	N	Y	Y	(7)High
Johansson et al. (2012)	Bus	N	Y	Y	N	Y	N	N	Y	N	(4)Low
Krause et al. (2004)	Bus	Y	Y	Y	N	Y	Y	N	Y	N	(6)Med
Krause et al. (1998)	Bus	Y	Y	Y	N	Y	Y	N	Y	Y	(7)High
Rugulies and Krause (2005)	Bus	Y	Y	Y	N	Y	Y	N	Y	Y	(7)High
Rugulies and Krause (2008)	Bus	Y	Y	Y	N	Y	Y	N	Y	Y	(7)High
Schwarze et al. (1998)	Truck & forklift	Y	Y	Y	N	Y	Y	N	Y	Y	(7)High
Tiemessen et al. (2008)	Bus	Y	Y	N	Y	Y	Y	N	Y	N	(5)Med

*Note.* N = no; Y = yes; High = high quality (low risk of bias); Med = medium quality (moderate risk of bias); Low = low quality (high risk of bias); 1 – Representativeness of the exposed cohort. 2 – Selection of the nonexposed cohort. 3 – Ascertainment of exposure. 4 – Demonstration that outcome of interest was not present at the start of study. 5 – Study controls for important factor. 6 – Study controls for an additional factor. 7 – Assessment of outcome. 8 – Was follow-up long enough for outcomes to occur? 9 – Adequacy of follow-up cohorts.

**Table 6:** Associations Between Physical Risk Factors and WRMSDs Among Professional Drivers

Risk factor (Number of Studies)	Author (Date)	Quality	Protective	Strength of Relationship		
				Weak	Moderate	Strong
WBV exposure (*19*)	Boshuizen et al. (1990)	Med			OR 2.8 (1.6–5)	
	Boshuizen et al. (1992)	Med		OR 2.4 (1.3–4.2)		
	Bovenzi (2009)	High		OR 1.60 (0.9–2.9)		
	Bovenzi (2010)	High			OR 2.8 (1.3–5.9)	
	Bovenzi (2015)	High		OR 1.3 (1.0–1.7)		
	Bovenzi and Zadini (1992)	Med				OR 4.0 (1.8–9.3)
	Bovenzi and Betta (1994)	Low		OR 2.39 (1.6–3.7)		
	Bovenzi et al. (2006)	High			OR 2.8 (1.3–6.0)	
	Burdorf et al. (1993)	Med		t-test (*p* < .05)		
	Gangopadhyay and Dev (2012)	Med				OR 4.9 (1.6–14.6)
	Magnusson et al. (1996)	Low		OR 2.0 (1.0–4.1)		
	Okunribido et al. (2008)	Med		Dose relationship (*p* < .05)		
	Palmer et al. (2008)	Med		OR 1.8 (0.8–4.1)		
	Robb and Mansfield (2007)†	Med		t-test (*p* < .01)		
	Schwarze et al. (1998)	High		RR 1.4 (0.9–2.2)		
	Sekkay et al. (2018)				OR 2.94 (1.36–6.34)	
	Tamrin et al. (2007)	Med		OR 1.9 (1.4–2.7)		
	Tamrin et al. (2014)	High			OR 3.2 (1.2–8.3)	
	Tiemessen et al. (2008)	Med			OR 3.5 (1.7–7.2)	
No association (1)	Okunribido et al. (2006)					
Awkward postures (*12*)	Bovenzi, Schust, et al. (2015)	High		OR 1.6 (1.0–2.4)		
	Bovenzi (2009)	High		OR 1.7 (1.2–2.6)		
	Bovenzi (2015)	High		OR 1.8 (1.2–2.9)		
	Bovenzi and Zadini (1992)	Med		OR 2.0 (1.1–3.8)		
	Bovenzi et al. (2006)	High		OR 1.7 (1.2–2.6)		
	Chen et al. (2004)	Med		OR 1.8 (1.1–2.8)		
	Chen et al. (2005)	Med		OR 1.8 (1.6–3.0)		
	Krause et al. (2004)	High		HR 1.7 (1.1–2.5)		
	Krause, Ragland, Greiner, Fisher, et al. (1997)	Med			OR 3.2 (2.1–5.1)	
	Palmer et al. (2008)	Med		OR 1.8 (1.1–2.9)		
	Szeto and Lam (2007)	Med		OR 2.2 (1.5–3.3)		
	Tiemessen et al. (2008)	Med		OR 1.7 (1.2–2.6)		
No association (*4*)	Okunribido et al. (2008, 2007); Tamrin et al. (2014, 2007)					
Static postures (*2*)	Szeto and Lam (2007)	Med			OR 3.7 (2.4–5.7)	
	Tamrin et al. (2014)	High				OR 6.1 (2.2–6.8)
No association (*2*)	Bovenzi (2009); Tiemessen et al. (2008)					
Lifting tasks (*9*)	Abledu et al. (2014b)	Med		OR 2.4 (1.4–5.7)		
	Bovenzi (2009)	High		OR 1.4 (0.8–2.2)		
	Bovenzi (2015)	High		OR 1.4 (0.9–2.2)		
	Bovenzi et al. (2006)	High		OR 1.9 (1.2–3.1)		
	Magnusson et al. (1996)	Low		OR 1.9 (1.2–2.8)		
	Robb and Mansfield (2007)	High		t-test (*p* < .05)		
	Tamrin et al. (2007)	Med		OR 1.2 (0.7–2.2)		
	Tamrin et al. (2014)	High		OR 2.4 (0.6–10)		
	Tiemessen et al. (2008)	Med		OR 1.7 (1.2–2.6)		
No association (2)	Okunribido et al. (2008); Palmer et al. (2008)					
Bending and twisting / turning (*4)*	Bovenzi (2009)	High		OR 1.51 (1.08–2.09)		
	Chen et al. (2004)	Med		OR 1.75 (1.09, 2.80)		
	Chen et al. (2005)	Med		OR 1.86 (1.15–3.00)		
	Bovenzi (2015)	High		OR 1.84 (1.19 – 2.85)		
No association	Nil					
Manual labor/Manual material handling *(3)*	Okunribido et al. (2006)	Med		OR 1.60(0.52–5.4)		
	Krause et al. (1998)	High			OR 3.04(1.85–5.00)	
	Sekkay et al. (2018)	High			OR 2.58 (1.21–5.49)	
No association (3)	Okunribido et al. (2008); Tamrin et al. (2014); Robb and Mansfield (2007); Raanaas and Anderson (2008)					

*Note.* HR = hazard ratio; OR = odds ratio; RR = risk ratio; WBV = whole-body vibration; WRMSDs = work-related musculoskeletal disorders.

### Psychosocial Risk Factors

There was moderate evidence of a weak relationship between WRMSDs and perceived job stress. Of 10 studies reviewed, nine were consistent in demonstrating a positive relationship with ORs ranging from 1.1 to 3.6. There was strong evidence of a weak relationship between WRMSDs and perceived job stress. Of nine studies reviewed, eight were consistent in demonstrating a positive relationship with ORs ranging from 1.3 to 3.4. Moderate evidence was found of a weak relationship between WRMSDs and low job satisfaction. Table 7 shows the associations between psychosocial RFs and WRMSDs with the strength of relationship.

**Table 7:** Associations Between Psychosocial Risk Factors and WRMSDs Among Professional Drivers

Risk Factor (*Number of Studies*)	Author (Date)	Quality	Strength of Relationship			
			Protective	Weak	Moderate	Strong
Perceived job stress (*9*)	Abledu et al. (2014a)	Med			OR 3.6 (1.6–8.2)	
	Abledu et al. (2014b)	Med			OR 2.7 (1.8–3.9)	
	Alperovitch-Najenson, Santo, et al. (2010)	Med		OR 1.6 (1.0–2.6)		
	Bovenzi (2015)	High		OR 2.1(1.3–3.6)		
	Chen et al. (2004)	Med			OR 2.5 (1.6–3.8)	
	Chen et al. (2005)	Med		OR 2.2 (1.6–3.0)		
	Greiner and Krause (2006)	High		OR 1.6 (1.2–2.0)		
	Tamrin et al. (2007)	Med		OR 1.1 (1.0–1.1)		
	Tamrin et al. (2014)	High			OR 3.0 (2.0–4.3)	
No association (*2*)	Alperovitch-Najenson, Katz-Leurer, et al. (2010); Krause et al. (1998)					
Perceived job demand (8)	Alperovitch-Najenson, Santo, et al. (2010)	Med		OR 1.6 (1.0–2.6)		
	Anjomshoae and Abdul Rani (2013)	Low		X2 test (*p* < .05)		
	Burdorf et al. (1993)	Med			OR 3.4 (1.3–9)	
	Burgel and Elshatarat (2017)	Low	OR .7 (0.5–0.9)			
	Gangopadhyay and Dev (2012)	Low			OR 3.3 (1.6–7.0)	
	Krause, Ragland, Greiner, Syme, et al. (1997)	Med		OR 2.0 (1.3–3.1)		
	Krause et al. (1998)	High		OR 1.5 (1.3–2.0)		
	Rugulies and Krause (2005)	High		HR 1.3 (0.9–2.1)		
No association (*1*)	Alperovitch-Najenson, Katz-Leurer, et al. (2010).					
Low job satisfaction (*8*)	Abledu et al. (2014a)	Med				OR 4.5 (1.7–11.7)
	Abledu et al. (2014b)	Med		OR 2.3 (1.6–4.0)		
	Bovenzi (2009)	High		OR 1.6 (0.8–3.4)		
	Bovenzi (2015)	High		OR 1.9 (1.0–3.7)		
	Chen et al. (2004)	Med		OR 1.5 (1.1–2.1)		
	Chen et al. (2005)	Med		OR 1.5 (1.1–2.0)		
	Krause et al. (1998)	High		OR 1.6 (1.1–2.2)		
	Rugulies and Krause (2008)	Med		HR 1.1 (1.0–1.3)		
No Association (*4*)	Bovenzi et al. (2006); Gyi and Porter (1998); Palmer et al. (2008); Tiemessen et al. (2008)					
Effort reward imbalance *(2)*	Rugulies and Krause (2008)	High		HR 1.32 (0.94 to 1.86)		
	Sekkay et al. (2018)	High		OR 2.35 (0.96–5.76)		
No association	Nil					

*Note.* HR = hazard ratio; OR = odds ratio; WRMSDs = work-related musculoskeletal disorders.

### Individual Risk Factors

Table 8 shows the studies that had reported individual RFs and their relationship with WRMSDs. Inconclusive evidence was observed for a weak relationship between WRMSDs and age of professional drivers. Of 31 reviewed studies, four presented a relationship with WRMSDs (age ranges not reported). There was inconclusive evidence supporting a weak relationship between WRMSDs and weight/BMI of professional drivers. Of 27 reviewed studies, eight studies presented a relationship with 19 reporting no association. An inconclusive evidence of a weak relationship was found between lack of physical activity/sports activity and WRMSDs with nine studies out of 19 showing a relationship; however, 10 reported no association. Strong evidence of a strong relationship between WRMSDs and previous musculoskeletal pain episodes was found. All eight reviewed studies demonstrated a positive relationship with ORs ranging from 2.2 to 5.4. There were moderate to strong evidence of a weak relationship between smoking, alcohol, gender (female), and WRMSDs among professional drivers.

**Table 8:** Associations Between Individual Risk Factors and WRMSDs with the Strength of Relationship

Risk Factor (*Number of Studies*)	Author (Date)	Quality	Strength of Relationship			
			Protective	Weak	Moderate	Strong
Age (*4*)	Aminian et al. (2016)	Med		OR 1.1 (1.0–1.1)		
	Bovenzi (2009)	High		OR 2.0 (1.0–4.0)		
	Bovenzi and Betta (1994)	Low			OR 3.4 (2.5–4.7)	
	Mozafari et al. (2015)	Med		X2 test *p* < .001		
No association (*27*)	Abledu et al. (2014a); Abledu et al. (2014b); Akinpelu et al. (2011); Alperovitch-Najenson, Katz-Leurer, et al. (2010); Alperovitch-Najenson, Santo, et al. (2010); Andrusaitis et al. (2006); Bovenzi et al. (2006); Bovenzi (2010, 2015); Bovenzi and Zadini (1992); Burdorf et al. (1993); Burgel and Elshatarat (2017); Gyi and Porter (1998); Krause et al. (2004); Krause et al. (1998); Krause, Ragland, Greiner, Fisher, et al. (1997); Krause, Ragland, Greiner, Syme, et al. (1997); Magnusson et al. (1996); Miyamoto et al. (2008, 2000); Okunribido et al. (2006, 2008); Okunribido et al. (2007); Palmer et al. (2008); Raanaas and Anderson (2008); Szeto and Lam (2007); Wang et al. (2017)					
Weight/BMI ()	Bovenzi et al. (2006)	High		OR 1.9 (1.1–3.3)		
	Bovenzi and Zadini (1992)	Med		OR 1.3 (0.7–2.6)		
	Mozafari et al. (2015)	Med		X2 test *p* < .001		
	Okunribido et al. (2006)	Med		X2 test *p* < .05		
	Palmer et al. (2008)	Med		OR 1.5 (0.9–2.5)		
	Raanaas and Anderson (2008)	High		OR 1.4 (1.0–1.9)		
	Tamrin et al. (2014)	High		OR 1.1 (0.8–1.5)		
	Wang et al. (2017)	High		OR 1.8 (1.1–2.8)		
No association (*19*)	Aminian et al. (2016); Anderson (1992); Andrusaitis et al. (2006); Bovenzi (2009, 2010, 2015); Bovenzi and Betta (1994); Burdorf et al. (1993); Gyi and Porter (1998); Krause et al. (1998, 2004); Krause, Ragland, Greiner, Syme, et al. (1997) ; Magnusson et al. (1996); Miyamoto et al. (2008, 2000); Okunribido et al. (2008); Okunribido et al. (2007); Rugbeer et al. (2016); Tiemessen et al. (2008)					
Lack of physical/sports activity (*7*)	Abledu et al. (2014a)	Med			OR 3.6 (1.4–9.3)	
	Abledu et al. (2014b)	Med			OR 3.2 (1.4–3.8)	
	Alperovitch-Najenson, Santo, et al. (2010)	Med		OR 2.1 (1.4–3.3)		
	Bovenzi and Betta (1994)	Low		OR 1.5 (1.1–2.1)		
	Chen et al. (2004)	Med		OR 1.8 (1.1–2.8)		
	Geete et al. (2013)	Low		OR 1.7 (1.0–2.6)		
	Raanaas and Anderson (2008)	Med		OR 2.0 (1.2–3.2)		
No association (*10*)	Alperovitch-Najenson, Katz-Leurer, et al. (2010); Anderson (1992); Andrusaitis et al. (2006); Bovenzi (2009, 2010); Bovenzi et al. (2006); Bovenzi and Zadini (1992); Gyi and Porter (1998); Magnusson et al. (1996); Okunribido et al. (2008)					
Previous musculoskeletal pain episodes (*8*)	Bovenzi and Betta (1994)	Med			OR 2.5 (1.7–3.5)	
	Bovenzi (2010)	High				OR 4.3 (not available)
	Bovenzi, Schust, et al. (2015)	High				OR 3.8 (2.2–6.6)
	Bovenzi and Betta (1994)	Low		OR 2.2 (1.5–3.1)		
	Gyi and Porter (1998)	Med		X2 test (*p* < .05)		
	Miyamoto et al. (2008)	Med				OR 5.4 (3.9–7.4)
	Okunribido et al. (2008)	Med				OR 4.1 (2.5–6.8)
	Robb and Mansfield (2007)	High		t-test (*p* < .001)		
No association (21)	Aminian et al. (2016); Anderson (1992); Andrusaitis et al. (2006); Bovenzi (2009, 2010, 2015)*; Bovenzi and Betta (1994); Burdorf et al. (1993); Gyi and Porter (1998); Krause et al. (2004); Krause et al. (1998)*; Krause, Ragland, Greiner, Fisher, et al. (1997); Krause, Ragland, Greiner, et al. (1997); Magnusson et al. (1996); Miyamoto et al. (2008, 2000); Okunribido et al. (2008); Okunribido et al. (2007); Rugbeer et al. (2016); Tiemessen et al. (2008)*					
Smoking *(5)*	Tamrin et al. (2014)	High		OR 1.5 (1.12, 2.14)		
	Palmer et al. (2008)	Med		OR 1.9 (1.2–3.0)		
	Miyamoto et al. (2008)	Med		OR 1.78 (1.29–2.45)		
	Bovenzi et al. (2006)	High		OR .79 (0.55–1.13)		
	Bovenzi and Betta (1994)	Low		OR 1.0 (0.75–1.31)		
No association (8)	Wang et al. (2017); Porter and Gyi (2002); Okunribido et al. (2006); Miyamoto et al. (2000); Bovenzi (2009, 2010); Andrusaitis et al. (2006); Abledu et al. (2014b)					
Alcohol *(3)*	Sekkay et al. (2018)	High			OR 2.78 (1.03–7.47)	
	Okunribido et al. (2006)	Med		OR 1.0 (0.5–1.9)		
	Bovenzi et al. (2006)	High		OR 1.24 (0.88–1.74		
No association (4)	Abledu et al. (2014a); Bovenzi, Schust, et al. (2015); Wang et al. (2017); Abledu et al. (2014b)					
Education *(3)*	Aminian et al. (2016)	Med		OR 2.07 (1.18–3.61)		
	Bovenzi and Zadini (1992)	High		OR 1.94(1.06–3.54)		
	Bovenzi and Betta (1994)	Low		OR 1.35 (0.95–1.84)		
No association (9)	Abledu et al. (2014a, 2014b); Bovenzi, Schust, et al. (2015); Burgel and Elshatarat (2017); Senthanar and Bigelow (2018); Wang et al. (2017); Bovenzi (2009, 2010); Bovenzi et al. (2006)					
Gender *(5)*	Raanaas and Anderson (2008)	High			OR 2.57 (1.63–4.06)	
	Szeto and Lam (2007)	Med		OR 1.78 (1.03–3.06)		
	Okunribido et al. (2006)	Med			OR 2.7 (1.0–7.7)	
	Krause, Ragland, Greiner, Fisher, et al. (1997)	Med		OR 2.14 (1.33–3.44)		
	Krause et al. (2004)	Med		OR 2.02 (1.15–3.55)		
No association (2)	Burgel and Elshatarat (2017); Krause et al. (1998)					

*Note.* BMI = body mass index; OR = odds ratio; WRMSDs = work-related musculoskeletal disorders.

### Other Risk Factors

The review provided inconclusive evidence between WRMSDs and years of driving occupation. Among the 23 studies reviewed on the years of professional driving experience, 11 demonstrated a positive relationship while 12 studies reported no association. There is inconclusive evidence of an association between WRMSDs and driving duration. Of 28 studies reviewed, 18 studies demonstrated a positive relationship with ORs ranging from 1.1 to 3.3. However, 10 studies (including two of high quality) found no relationship. A strong evidence for a weak relationship was observed between decision latitude and WRMSDs. The category of other RFs showing association with WRMSDs with the strength of relationship is shown in Table 9.

**Table 9:** Other Risk Factors Showing Association With WRMSDs With the Strength of Relationship

Risk Factor (Number of Studies)	Author (Date)	Quality	Protective	Strength of Relationship		
				Weak	Moderate	Strong
Years in driving occupation (*11*)	Abledu et al. (2014a)	Med		OR 1.8 (0.6–5.2)		
	Anderson (1992)	Med		X^2^ test *p* < .05		
	Akinpelu et al. (2011)	Med		X^2^ test *p* < .05		
	Boshuizen et al. (1992)	Med			OR 3.6(1.2–11)	OR 5.7 (1.04–31)
	Krause, Ragland, Greiner, Fisher, et al. (1997)	Med			<5 years OR 3.4 (1.5–7.8)	
	Krause et al. (1998)	High	>15 years OR .5 (0.3–0.7)			>5 years OR 6.1 (4.1–9.1)
	Krause et al. (2004)	High		<5 years HR 1.4 (1.0–1.8)		
	Porter and Gyi (2002)	High		r = .02 (*p* < .05)		
	Raanaas and Anderson (2008)	High	OR .6 (0.5–0.9)			
	Szeto and Lam (2007)	Med		OR 2.5 (1.4–4.5)		
	Wang et al. (2017)	High		OR 1.7 (1.2–2.5)		
No association (12)	Abledu et al. (2014b); Andrusaitis et al. (2006); Bovenzi et al. (2006); Burdorf et al. (1993); Burgel and Elshatarat (2017); Greiner and Krause (2006); Miyamoto et al. (2008, 2000); Okunribido et al. (2007); Palmer et al. (2008); ; Tamrin et al. (2014, 2007).					
Driving duration (hours per day/week) *(18*)	Abledu et al. (2014a)	Med			OR 3.3 (1.2–8.5)	
	Abledu et al. (2014b)	Med			OR 2.8 (1.2–8.5)	
	Aminian et al. (2016)	Med		OR 1.1 (1.0–1.03)		
	Andrusaitis et al. (2006)	Med		OR 1.1 (1.0–1.1)		
	Bovenzi (2010)	High			OR 2.8 (0.9–9.0)	
	Chen et al. (2004)	Med		OR 2.1 (1.1–3.9)		
	Chen et al. (2005)	Med		POR 2.1 (1.3–3.6)		
	Gangopadhyay and Dev (2012)	Med			OR 3.3 (1.6–7)	
	Gyi and Porter (1998)	Med		r = .01 (*p* < .05)		
	Johansson et al. (2012)	Low		LR *p* < .001		
	Krause, Ragland, Greiner, Fisher, et al. (1997)	Med		OR 2.0(1.0–3.6)		
	Krause et al. (2004)	High		HR 1.4 (1.2–1.7)		
	Miyamoto et al. (2000)	Low		OR 2.0 (1.0–4.3)		
	Mozafari et al. (2015)	Med		X^2^ test *p* < .001		
	Raanaas and Anderson (2008)	Med		OR 2.0 (1.1–3.6)		
	Robb and Mansfield (2007)	Med		t-test (*p* < .01)		
	Tamrin et al. (2014)	High		OR 1.3 (1.0–1.8)		
	Wang et al. (2017)	High			OR 3.3 (1.9–5.9)	
No association (10)	Akinpelu et al. (2011); Anderson (1992); Bovenzi et al. (2006); Burgel and Elshatarat (2017); Greiner and Krause (2006); Miyamoto et al. (2008); Okunribido et al. (2007); Palmer et al. (2008); Rugbeer et al. (2016); Tamrin et al. (2007)					
Uncomfortable seat (*8*)	Alperovitch-Najenson, Katz-Leurer, et al. (2010)	Med			OR 2.6 (1.4–5.0)	
	Alperovitch-Najenson, Santo, et al. (2010)	Med		OR 2.2 (1.2–4.2)		
	Gangopadhyay and Dev (2012)	Low			OR 3.3 (1.4–7.0)	
	Geete et al. (2013)	Low			OR 3.2 (1.6–5.2)	
	Okunribido et al. (2008)	Med				OR 4.4 (2.7–7.1)
	Rugbeer et al. (2016)	Med		r = .25 (*p* < .05)		
	Szeto and Lam (2007)	Med		OR 2.21 (1.5–3.3)		
	Tamrin et al. (2014)	High			OR 3.4 (1.9–5.9)	
No association (*1*)	Okunribido et al. (2006)					
Sleep *(2)*	Wang et al. (2017)	High	OR .7 (0.5–0.9)			
	Miyamoto et al. (2008)	Med		OR 2.21 (1.61–3.01)		
No association (3)	Sekkay et al. (2018); Miyamoto et al. (2000); Raanaas and Anderson (2008)					
Decision latitude *(4)*	Sekkay et al. (2018)	High			OR 2.73 (1.07–6.93)	
	Palmer et al. (2008)	Med		OR 1.8 (1.1–2.8)		
	Bovenzi et al. (2006)	High		OR 1.14 (0.70–1.84)		
	Bovenzi, Schust, et al. (2015)	High		OR 1.82 (1.07– 3.09)		
No association (3)	Krause et al. (1998); Rugulies and Krause (2005); Bovenzi (2009)					

*Note.* HR = hazard ratio; OR = odds ratio; WRMSDs = work-related musculoskeletal disorders

## Discussion

The aim of this systematic review was to identity and evaluate the relationship between RFs and WRMSDs among professional drivers. Establishing causal links between WRMSDs and associated RFs from single studies would be unreliable due to limitations imposed by specific study designs and populations, methodological quality, and specific types of statistical analysis (da Costa & Vieira, 2010). Therefore, the current review extracted RFs from multiple studies in order to gain a comprehensive understanding of their relationship with WRMSDs. In summary, the findings of the review identified a total of 23 different types of RFs (physical: 6, psychosocial: 4, individual: 8, and other: 5) related to WRMSDs from a collection of 54 studies. In general, several physical RFs showed a strong evidence of a relationship with WRMSDs. Moderate to strong evidence of a relationship was observed among psychosocial factors, while previous episodes of the musculoskeletal pain were the only individual risk factor that showed a strong evidence of relationship with WRMSDs. Other RFs such as tenure and duration of driving showed inconclusive evidence. Although various RFs showed relationship with WRMSDs among professional drivers, the overall strength of the relationship was generally weak across the findings.

While the strength of the study was the comprehensive approach in evaluating the relationship between RFs and WRMSDs among professional drivers, it was also the weakness as it added to the heterogeneity of the studies included. The review included studies that presented different types of professional drivers who were driving different types of vehicles such as bus, truck, car/taxi, minivan, crane, forklift, and so on. It was possible that the working conditions and physical factors might be very different among the drivers reported in the studies. For instance, while some drivers may operate heavy vehicles in off-road conditions, other drivers such as bus and taxi drivers work on road conditions. Further, different types of drivers might have different job demands with some drivers involved in lifting heavy objects dealing with high force of exertion and others might not do such heavy manual tasks. In addition, different sizes of the vehicles meant a different size of operating space for drivers, which might impact their posture and WBV. For example, drivers working with vehicles such as farm tractors and heavy machinery vehicles might need to work with twisted torso and posture as opposed to bus drivers. Also, exposure of the drivers to vibration might vary, demonstrating a larger range in terms of magnitude and daily duration. Furthermore, the current review did not account for the magnitude of the exposure among the drivers due to the wide range of reporting standards of the vibration and years of exposure among the studies. The above variations might explain why some of the acknowledged exposure factors such as time/years of driving and vibration showed no relationship in this review with WRMSDs. Therefore, all the above factors might have contributed to some unmeasured biases in the results; hence, they need to be considered as potential confounding factors while interpreting the review findings. Thus, it is possible that the potential impact of the above variability might explain why no association was found for some of the RFs.

This review was different from previous systematic reviews as it evaluated the evidence on the strength of causal relationship between categories of RFs and WRMSDs among professional drivers using the Bradford–Hill causation criteria. Causal inference is referred to as a scientific process that tests whether a relation of cause to effect exists (Susser, 2001). However, the assessment of causality in health conditions is a challenging process as most diseases including WRMSDs have a multifactorial origin and pathogenesis (Doll, 2002). In this review, the Bradford–Hill causation criteria were used to interpret the causation logically rather than empirically. While the Bradford–Hill criteria in this review were not used as a rule to judge an association as causal, it served as a reasonable inference to examine the cause and effect (Lucas & McMichael, 2005). Therefore, the criteria of causality used in this review must be viewed as an inferential judgment rather than arbiters of reality (Lucas & McMichael, 2005). Another important challenge reported in the social and health sciences relates to making justified causal inferences using nonexperimental, observational data (Ward, 2009). Epidemiological studies are generally observational and nonexperimental conducted in a natural environment among free living population (Lucas & McMichael, 2005). In other words, the inclusion of epidemiological studies in this review meant that there might be several other independent factors that could influence the exposure mechanism and disease outcome. Nevertheless, in order to determine the risk of WRMSDs in occupational settings, epidemiological studies must be performed. Practitioners and policy makers are encouraged to consider the above-said challenges related to causation criteria while engaging with the current review findings.

Several gaps and issues exist around the policies and clinical practices toward effective management of RFs related to WRMSDs. The clinical practice and existing policies on WRMSDs largely fail to address RFs as a source of the problem (Oakman & Chan, 2015, Oakman & Macdonald, 2019). Any interventions for WRMSDs should not be based merely on addressing the symptoms, but must also focus in managing the significant RFs that are associated with WRMSDs. However, the current risk management practices at workplaces fail to address risk reduction for musculoskeletal disorders comprehensively and effectively (Oakman et al., 2018). The Department of Health and Social Care working with the Public Health England and Department for Work and Pensions has launched a 5-year strategic framework on improving musculoskeletal health for the public and recommended a logical model to support good practice of musculoskeletal health at the workplace (Department of Health and Social Care Working with Public Health England and Department for Work and Pensions, 2019). However, there were no explicit details available on RFs for WRMSDs for occupations like professional driving. Similarly, the United Kingdom Health and Safety Executive policy documents on the risk assessment clearly cover the driver’s safety at the work; however, an explicit assessment and management of RFs relating to WRMSDs among the drivers are lacking (Health & Safety Executive [HSE], 2020). While the Canadian Center of Occupational Health and Safety has provided information on RFs, this information is very general and targeted specific information of RFs on certain occupations such as professional drivers is clearly lacking (Canadian Centre for Occupational Health and Safety, 2020).

In the light of the above-stated gaps and issues regarding RFs related to WRMSDs, the findings of the current review might contribute to the planning, decision-making, and actions of policy makers, researchers, and occupational musculoskeletal health practitioners. The demonstrated strong evidence of the relationship between physical and psychosocial factors and WRMSDs might suggest the need for policy makers to develop policies considering a multifactorial approach to screen and evaluate physical-psychosocial RFs rather than addressing individual specific hazards associated with WRMSDs among professional drivers. Perhaps, both physical and psychosocial influences as a holistic approach to work environments should be considered in any policy development and management strategies. The inconclusive evidence on the individual RFs means that perhaps addressing individual factors might not be an effective approach to manage WRMSDs among professional drivers. In some of the physical RFs, such as WBV and awkward posture that showed a strong evidence of association with WRMSDs, it might be helpful to make more specific information available in terms of what awkward postures and what magnitudes of the WBV were associated with WRMSDs. Such information could provide more concrete and strategic approaches for the prevention and management of WRMSDs. The relationship between multiple factors and WRMSDs in professional drivers warrants researchers to develop an effective multifactorial toolkit to evaluate RFs. Therefore, further research is required for the development of valid assessment tools to evaluate RFs among professional drivers. As such, the review findings provided an insight to the SMART Drive network partners to design and develop a preliminary proforma to evaluate the multiple RFs for WRMSDs among professional drivers.

### Strength and Limitations

The review has some limitations. A wide heterogeneity of the parameters in the included studies was one of the limitations. This could influence the translation of the review findings into practice and therefore should be interpreted with caution. For the continuous variables, the values for strength of relationship of the logistic regressions, the ORs, and the relative RRs may depend on the dimensions of the exposure (risk factor) being examined. The different scales of exposures for the continuous variables across different studies were not adjusted due to heterogeneity. Instead, the reported values of logistic regression, the ORs, and the relative RRs reported in the studies were directly extracted and interpreted. Thus, in spite of the strength of relationship, the exposure metrics of some RFs and their dimensions vary and therefore may weaken the evidence. The relationship of some of the factors (e.g., diet, activities above the shoulder level, and repetitive movements) with WRMSDs could not be estimated due to lack of data reported among the studies. Also, it was possible that the variability of a specific exposure in some studies of certain driver populations was not wide enough, and therefore association could not be found. A wide heterogeneity was also noticed in phrasing some RFs across the studies. For example, the studies had used and reported different phrases such as “lifting tasks, push-pulling activities, carrying things, manual materials handling, and activities above the shoulder level,” without providing a clear definition of these activities. This made it difficult to interpret and classify these RFs. It is therefore recommended that clear standard operational definitions and terminology of the occupational tasks be developed for the use in research as well as in professional practice. A meta-analysis on the causal relationship between RFs and WRMSDs was not possible due to a wide heterogeneity of outcome measures reported among the reviewed studies. Nevertheless, the review was conducted according to the good standards of practice recommended by PRISMA guidelines (Moher et al., 2015; Liberati et al., 2009). Also, well-established Bradford–Hill criteria for causation were used to evaluate the strength of the relationship between WRMSDs and the associated RFs (Roffey et al., 2010; Rosenthal, 1996). Thus, the current review employed a strong method by summarizing statistical findings from different studies and presented a logical interpretation of RFs and their relationship to WRMSDs in the population of professional drivers. Another limitation was that the review included studies only in the English language, which could have introduced a language publication bias. However, as English was the common language among the SMART Drive network partners, it was decided to include articles published in English only. However, as can be seen in the titles of the reviewed articles, many of the studies were conducted in non-English-speaking countries, which gives some confidence that the topic is acute and applicable across the world. Further evaluation and comparison of the differences and similarities in different countries were outside the scope of this study. With only a limited number of good-quality prospective cohort studies (10 studies) available for this review, one might argue that the evidence for reporting causal relationships between WRMSDs and associated RFs might be weak. Additional high methodological quality prospective cohort studies are required for further understanding of the causal relationships between RFs and WRMSDs among professional drivers.

## Conclusion

The findings showed evidence of the causal relationship between RFs and WRMSDs among professional drivers. The RFs with strong evidence of a relationship with WRMSDs include WBV, awkward postures, lifting tasks, perceived job stress, perceived job demand, and previous musculoskeletal pain episodes. RFs with moderate evidence of a relationship WRMSDs include uncomfortable seat and low job satisfaction. RFs with inconclusive evidence of a relationship with WRMSDs include years in professional driving and driving duration, age, and weight/BMI. As a conclusion, the review demonstrated that the physical, psychosocial, and individual factors all pose risks for WRMSDs among professional drivers. Therefore, holistic and multidisciplinary attention is required to develop prevention and management policies and strategies to address this common and multifaceted issue affecting a large section of the working population.

## Key Points

- This review suggests a causal relationship between physical, psychosocial, and individual risk factors and work-related musculoskeletal disorders among professional drivers
- Strong evidence exists on the causal relationship between physical risk factors (whole body vibration, awkward posture, lifting task) and musculoskeletal disorders
- Strong evidence supports the causal relationship between psychosocial risk factors (job stress, job demand, and previous pain episodes) and musculoskeletal disorders
- Moderate evidence suggests a causal relationship between uncomfortable seat and low job satisfaction with musculoskeletal disorders.
- The results of this review have implications for developing appropriate screening, monitoring, prevention, and management strategies of the risk factors related to work-related musculoskeletal disorders among professional drivers.

## Supplemental Material

Online supplementary file 1 - Supplemental material for Causal Relationship Between the Risk Factors and Work-Related Musculoskeletal Disorders Among Professional Drivers: A Systematic Review Click here for additional data file. Supplemental material, Online supplementary file 1, for Causal Relationship Between the Risk Factors and Work-Related Musculoskeletal Disorders Among Professional Drivers: A Systematic Review by Leonard Joseph, Lenny Vasanthan, Miles Standen, Raija Kuisma, Aatit Paungmali, Ubon Pirunsan and Patraporn Sitilertpisan in Human Factors: The Journal of Human Factors and Ergonomics Society
