# Supplementary material for: Causal Relationship Between the Risk Factors and Work-Related Musculoskeletal Disorders Among Professional Drivers: A Systematic Review
Source: Hum Factors. 2021 Jun 14;65(1):62–85. doi: 10.1177/00187208211006500 (PMC9846379; doi:10.1177/00187208211006500)
Supplement: Online supplementary file 1 - Supplemental material for Causal Relationship Between the Risk Factors and Work-Related Musculoskeletal Disorders Among Professional Drivers: A Systematic Review [file sj-docx-1-hfs-10.1177_00187208211006500.docx]

**SEARCH STRATEGY**

Specific search terms, search fields, combinations of terms and search results are presented.

1 exp automobile driving/ (22989)

2 professional driv*.mp. (609)

3 occupational driv*.mp. [mp=title, abstract, heading word, drug trade name, original title, device manufacturer, drug manufacturer, device trade name, keyword, floating subheading word] (72)

4 work-related driv*.mp. [mp=title, abstract, heading word, drug trade name, original title, device manufacturer, drug manufacturer, device trade name, keyword, floating subheading word] (19)

5 industrial driv*.mp. [mp=title, abstract, heading word, drug trade name, original title, device manufacturer, drug manufacturer, device trade name, keyword, floating subheading word] (2)

6 business driv*.mp. [mp=title, abstract, heading word, drug trade name, original title, device manufacturer, drug manufacturer, device trade name, keyword, floating subheading word] (46)

7 ambulance driv*.mp. [mp=title, abstract, heading word, drug trade name, original title, device manufacturer, drug manufacturer, device trade name, keyword, floating subheading word] (80)

8 bus driv*.mp. [mp=title, abstract, heading word, drug trade name, original title, device manufacturer, drug manufacturer, device trade name, keyword, floating subheading word] (685)

9 cab driv*.mp. [mp=title, abstract, heading word, drug trade name, original title, device manufacturer, drug manufacturer, device trade name, keyword, floating subheading word] (26)

10 coach driv*.mp. [mp=title, abstract, heading word, drug trade name, original title, device manufacturer, drug manufacturer, device trade name, keyword, floating subheading word] (12)

11 delivery driv*.mp. [mp=title, abstract, heading word, drug trade name, original title, device manufacturer, drug manufacturer, device trade name, keyword, floating subheading word] (41)

12 lorry driv*.mp. [mp=title, abstract, heading word, drug trade name, original title, device manufacturer, drug manufacturer, device trade name, keyword, floating subheading word] (100)

13 forklift driv*.mp. [mp=title, abstract, heading word, drug trade name, original title, device manufacturer, drug manufacturer, device trade name, keyword, floating subheading word] (13)

14 minibus driv*.mp. [mp=title, abstract, heading word, drug trade name, original title, device manufacturer, drug manufacturer, device trade name, keyword, floating subheading word] (5)

15 motorcar driv*.mp. [mp=title, abstract, heading word, drug trade name, original title, device manufacturer, drug manufacturer, device trade name, keyword, floating subheading word] (7)

16 racing driv*.mp. [mp=title, abstract, heading word, drug trade name, original title, device manufacturer, drug manufacturer, device trade name, keyword, floating subheading word] (35)

17 racecar driv*.mp. [mp=title, abstract, heading word, drug trade name, original title, device manufacturer, drug manufacturer, device trade name, keyword, floating subheading word] (10)

18 taxi driv*.mp. [mp=title, abstract, heading word, drug trade name, original title, device manufacturer, drug manufacturer, device trade name, keyword, floating subheading word] (431)

19 truck driv*.mp. [mp=title, abstract, heading word, drug trade name, original title, device manufacturer, drug manufacturer, device trade name, keyword, floating subheading word] (1156)

20 tractor driv*.mp. [mp=title, abstract, heading word, drug trade name, original title, device manufacturer, drug manufacturer, device trade name, keyword, floating subheading word] (163)

21 van driv*.mp. [mp=title, abstract, heading word, drug trade name, original title, device manufacturer, drug manufacturer, device trade name, keyword, floating subheading word] (30)

22 emergency vehicle driv*.mp. [mp=title, abstract, heading word, drug trade name, original title, device manufacturer, drug manufacturer, device trade name, keyword, floating subheading word] (3)

23 police driv*.mp. [mp=title, abstract, heading word, drug trade name, original title, device manufacturer, drug manufacturer, device trade name, keyword, floating subheading word] (37)

24 exp musculoskeletal disease/ (2022474)

25 musculoskeletal disorder*.mp. [mp=title, abstract, heading word, drug trade name, original title, device manufacturer, drug manufacturer, device trade name, keyword, floating subheading word] (8013)

26 musculoskeletal symptom*.mp. [mp=title, abstract, heading word, drug trade name, original title, device manufacturer, drug manufacturer, device trade name, keyword, floating subheading word] (2242)

27 musculoskeletal discomfort.mp. [mp=title, abstract, heading word, drug trade name, original title, device manufacturer, drug manufacturer, device trade name, keyword, floating subheading word] (323)

28 musculoskeletal complaint*.mp. [mp=title, abstract, heading word, drug trade name, original title, device manufacturer, drug manufacturer, device trade name, keyword, floating subheading word] (1418)

29 cumulative trauma disorder*.mp. [mp=title, abstract, heading word, drug trade name, original title, device manufacturer, drug manufacturer, device trade name, keyword, floating subheading word] (2371)

30 musculoskeletal disease*.mp. [mp=title, abstract, heading word, drug trade name, original title, device manufacturer, drug manufacturer, device trade name, keyword, floating subheading word] (29337)

31 exp pain/ (1114680)

32 pain.mp. [mp=title, abstract, heading word, drug trade name, original title, device manufacturer, drug manufacturer, device trade name, keyword, floating subheading word] (1058173)

33 exp prevalence/ (592636)

34 prevalence.mp. [mp=title, abstract, heading word, drug trade name, original title, device manufacturer, drug manufacturer, device trade name, keyword, floating subheading word] (856300)

35 exp risk/ (2084898)

36 risk.mp. [mp=title, abstract, heading word, drug trade name, original title, device manufacturer, drug manufacturer, device trade name, keyword, floating subheading word] (3111569)

37 1 or 2 or 3 or 4 or 5 or 6 or 7 or 8 or 9 or 10 or 11 or 12 or 13 or 14 or 15 or 16 or 17 or 18 or 19 or 20 or 21 or 22 or 23 (25441) 38 24 or 25 or 26 or 27 or 28 or 29 or 30 or 31 or 32 (2911955)

39 33 or 34 or 35 or 36 (3649155)

40 37 and 38 and 39 (523)
